# Sex Differences in Dendritic Spine Formation in the Hippocampus and Animal Behaviors in a Mouse Model of Hyperthyroidism

**DOI:** 10.3389/fncel.2020.00268

**Published:** 2020-09-16

**Authors:** Tetsushi Niiyama, Mahomi Kuroiwa, Yusaku Yoshioka, Yosuke Kitahara, Takahide Shuto, Tatsuyuki Kakuma, Keisuke Ohta, Kei-ichiro Nakamura, Akinori Nishi, Mami Noda

**Affiliations:** ^1^Laboratory of Pathophysiology, Graduate School of Pharmaceutical Sciences, Kyushu University, Fukuoka, Japan; ^2^Department of Pharmacology, Kurume University School of Medicine, Kurume, Japan; ^3^Biostatistics Center, Kurume University, Kurume, Japan; ^4^Department of Anatomy, Kurume University School of Medicine, Kurume, Japan

**Keywords:** hyperthyroidism, dendritic spine, locomotor activity, depression, sex dependence

## Abstract

Thyroid hormones are critical for the regulation of development and differentiation of neurons and glial cells in the central nervous system (CNS). We have previously reported the sex-dependent changes of glial morphology in the brain under the state of hyperthyroidism. Here, we examined sex-dependent changes in spine structure of granule neurons in the dentate gyrus of hippocampus in male and female mice with hyperthyroidism. Using FIB/SEM (focused ion beam/scanning electron microscopy), three-dimensional reconstructed structures of dendritic spines in dentate granule cells were analyzed. Dendritic spine density in granule cells increased significantly in both male and female mice with hyperthyroidism. The decrease in spine volume was observed only in female mice. These findings suggest that hyperthyroidism induces the formation of spines with normal size in male mice but the formation of spines with small size in female mice. To evaluate an outcome of neuronal and previously observed glial changes, behavioral tests were performed. Male mice with hyperthyroidism showed increased locomotor activity in the open field test, while female mice showed elevated immobility time in the tail suspension test, reflecting depression-like behavior. Although direct link between changes in spine and behavioral modifications requires further analysis, our results may help to understand gender-dependent neurological and psychological symptoms observed in patients with hyperthyroidism.

## Introduction

The endocrine system and the nervous system are closely related with both neurons and glia contributing to the link between two systems (Chowen et al., 1996; Garcia-Segura et al., 1996; Noda, 2015; Morita et al., 2019). In the adult central nervous system (CNS), thyroid dysfunction triggers psychological conditions such as anxiety and depression (Paschke et al., 1990; Suwalska et al., 2005; Gulseren et al., 2006), and patients with attention-deficit/hyperactivity disorder have greater comorbid rates with hyperthyroidism (Chen et al., 2018; Zader et al., 2019). In the elderly, both hyperthyroidism and hypothyroidism potentially increase the risk of cognitive impairment and neurodegeneration including Alzheimer’s disease (Small et al., 1985; Kalmijn et al., 2000; Tan et al., 2008; de Jong et al., 2009; Tan and Vasan, 2009). We have reported the non-genomic effects of 3,3′,5-triiodothyronine (T3), an active form of thyroid hormone, on microglial functions *in vitro* (e.g., increased migration, membrane ruffling, and phagocytosis; Mori et al., 2015). Under *in vivo* conditions, administration of L-thyroxine (T4), which is taken up by astrocyte and converted into T3 by astrocyte-specific type 2-deiodinase (D2; Guadaño-Ferraz et al., 1997; Fliers et al., 2006; Di Liegro, 2008), induced the sex- and age-dependent effects on glial morphology (represented by morphological changes of microglia and astrocytes in young male mouse brain; Noda et al., 2016). In contrast, morphological changes in neurons remain to be characterized.

The effects of thyroid hormones on neuronal cells (Kollros and Pesetsky, 1956) and maturation of nervous tissue (Hamburgh et al., 1962) were reported since 1950. Until now, the critical roles of thyroid hormones in brain development and function as well as in regulation of gene expression are well documented (Bernal and Nunez, 1995; Bernal, 2015, 2017). Nevertheless, functions of thyroid hormone in adult brain and the contribution of thyroid dysfunction to depression (Joffe, 2011; Hage and Azar, 2012) are not fully understood while data available remain controversial.

Dentate gyrus plays a critical role in the pathophysiology of depression and mediates antidepressant action (Umschweif et al., 2019; Shuto et al., 2020). We previously reported that chronic antidepressant treatment induces the enlargement of perforant path-granule cell synapse in the dentate gyrus (Kitahara et al., 2016). We hypothesized that hyperthyroidism may affect the structure of dendritic spine in granule neurons in the dentate gyrus, this morphological remodeling being associated with depression-like phenotypes. Therefore, in this study, we analyzed the effects of thyroid hormones on the density and morphology of dendritic spines in the dentate gyrus and correlated these changes with behaviors in male and female mice. We found sex-independent increases in spine density and sex-dependent alterations of spine morphology and depression-like behaviors in an animal model of hyperthyroidism. These results may help to understand the physiological and/or pathophysiological functions of thyroid hormones in the CNS and reveal how hyperthyroidism affects psychological conditions in male and female.

## Materials and Methods

### Animal Care and Treatment

The study was approved by and conforms to the Guidelines of the Committee for Animal Care and Use of Kyushu University and Kurume University School of Medicine. Male and female C57BL/6N mice were purchased from Kyudo (Saga, Japan) and Japan SLC (Shizuoka, Japan). Hyperthyroidism was induced in the male and female mice at 10 weeks old by intraperitoneally injecting T4 (0.3 mg/kg, 10 mg/ml of 0.5 N NaOH and then diluted with phosphate buffered saline) four times in 2 weeks (at days 0, 4, 9, and 14; Noda et al., 2016). Control mice received the injection of the same solution without T4. At day 15, behavioral tests were performed and a different set of animals were subjected to the analyses of dendritic spines.

### Preparation of Dentate Gyrus Samples

The protocol was according to the method described previously (Kitahara et al., 2016). Briefly, mice were deeply anesthetized and then perfused with 2% paraformaldehyde and 2.5% glutaraldehyde in phosphate buffer (0.1 M, pH 7.4). Three to 4 h after perfusion, the brains were removed, and coronal slices of the dorsal dentate gyrus (DG; 300 μm) were cut with a vibrating blade microtome (VT1000S, Leica Microsystems, Nussloch, Germany). The slices were subsequently postfixed and *en bloc* stained for FIB/SEM. The slices were treated with 1% thiocarbohydrazide and were then immersed in a solution of 2% OsO_4_. For *en bloc* staining, the slices were immersed in a solution of 4% uranyl acetate solution overnight and washed with double distilled water. The slices were further stained with Walton’s lead aspartate solution, dehydrated in an ethanol series, placed in ice-cold dry acetone, subjected to infiltration of an epoxy resin (Epon 812, TAAB, England) mixture, and polymerized for 72 h at 60°C.

### Imaging With FIB/SEM

As described previously (Kitahara et al., 2016), the embedded slices were placed on a metal stub and further trimmed with glass and diamond knives in an ultramicrotome (Ultracut E microtome, Leica, Wetzlar, Germany). The slices were coated with a protective layer of carbons, and the metal stub with the slices was set on the stage of FIB/SEM. The serial section images in the middle molecular layer of the dorsal DG were automatically obtained by FIB/SEM (Quanta 3D FEG, FEI, Hillsboro, OR, USA). Serial images of the block face were acquired by repeated cycles of sample surface milling and imaging using the Slice and View G2 operating software (FEI). The milling was performed with a gallium ion beam at 30 kV with a current of 1.0 nA. The milling pitch was set at 30 nm/step. The images were acquired at an accelerating voltage of 2.5 kV. The other acquisition parameters were as follows: dwell time = 6 μs/pixel and pixel size = 4.9 nm/pixel.

### Three-Dimensional Reconstruction and Analysis of Spines

The serial section images obtained with FIB/SEM were reconstructed to 3D images and were analyzed using Amira 5.5 software (FEI), as reported previously (Kitahara et al., 2016). Structures of dendritic spine were manually traced, and the volume of spines was measured using Amira. For the analyses of dendritic spines, three dendrites were traced from each of three mice.

### Behavioral Tests

Animals were handled daily for 1 week before testing to adapt them to handling and introduce them to experimental room. Behavioral experiments were carried out in a semi-soundproof experimental room at least 30 min before each test. Any environmental or physical stress was avoided in order to habituate the mice to manipulation for behavioral tests. The open field test was performed first, and then about 1.5 h later, the tail suspension test was performed.

#### Open Field Test

To investigate the changes in spontaneous locomotor activity, grooming, rearing, and the time in the center circle, mice in all experimental groups were submitted to a 5-min period of the open field test. An apparatus for the open field test consisted of a round platform with a diameter of 60.0 cm and a wall height of 50.0 cm. The floor of the platform was divided into 19 areas by drawing lines radially and two inner circles. The platform was illuminated by a light source (lamp 60 W) at 80 cm height. Each mouse was placed in the center circle and allowed to freely explore the apparatus, with the experimenter out of the animal’s sight. Locomotor activity was measured by counting how many times the mouse crossed the line, the numbers of grooming and rearing with hind limbs were counted, and the time duration the mouse stayed in the central circle was measured. Two independent observers measured the behavioral variables. A video camera was installed above the platform to record the activity of the mice as well. After each test session, the platform was carefully cleaned by 70% ethanol.

#### Tail Suspension Test

The immobility was induced by suspending the mice by the tail (Castagné et al., 2009). The experimental room was equipped with white neon ceiling lights (standard lighting). Three quarters of the distance from the base of the mouse’s tail in a constant position was wrapped by adhesive rubber tape. Mice were suspended by passing the suspension hook through the adhesive tape so that the mice were hung with its tail in a straight line in a one-side-open box with 30 cm height, 23 cm width, and 22 cm depth. After initially trying to escape by engaging in vigorous movements, mice rapidly become immobile. The duration of immobility during 5 min was measured by a chronometer for each mouse. A video camera was installed for the behavioral tests for later analyses by independent observers.

### Statistical Analysis

The data are shown as the mean ± SEM. In statistical analyses of spine volume and structure, hypothesis test of two group means was carried out by two methods, because visual inspection of measurement distribution indicated that normality assumption of measurements is not tenable. First, Welch’s *t*-test allowing unequal variances was performed to obtain *p*-value. To examine robustness of the *p*-value, the bootstrap method (Efron and Tibshirani, 1993) was used to obtain the achieved significance level (ASLboot), which can be interpreted as *p*-value based on non-parametric Welch’s *t*-test. STATA/MP 16.0 (StataCorp, College Station, TX, USA) was used for these analyses. Statistical analyses for spine density and behaviors were performed using Welch’s *t*-test and Student’s *t*-test (GraphPad Prism, GraphPad Software, San Diego, CA, USA), respectively, as indicated in figure legends.

## Results

### Hyperthyroidism Affects Spine Density and Size Differently Between Male and Female Mice

**Figure 1:**
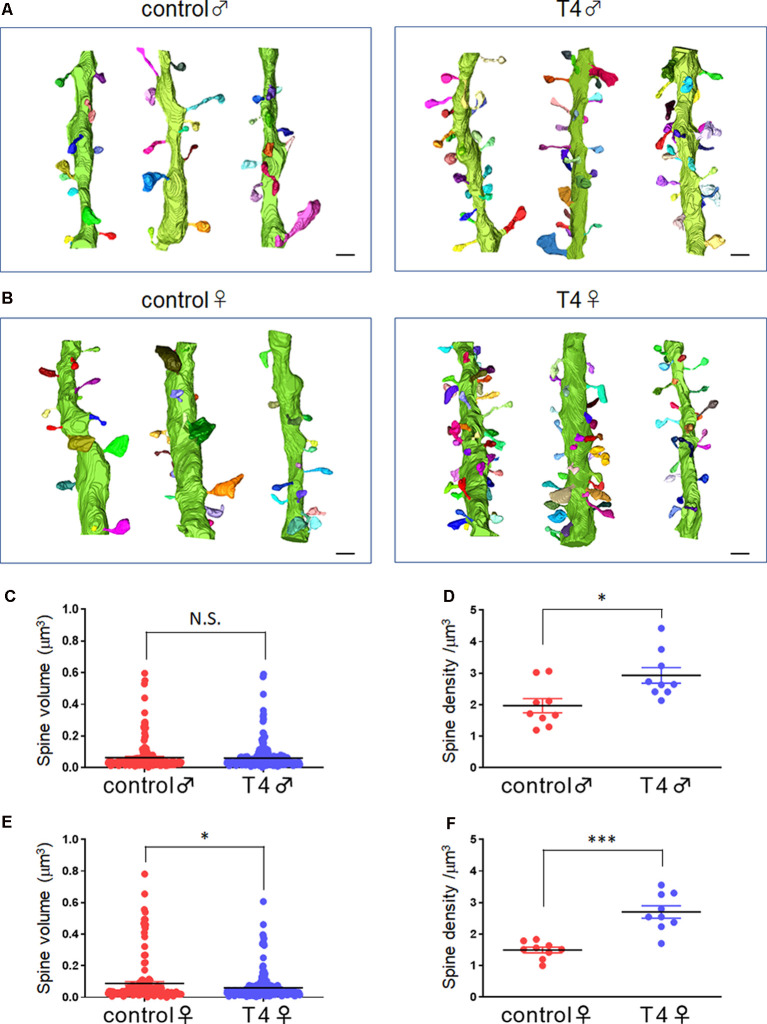
Spine morphology and density in hippocampal granule cells in male and female mice with hyperthyroidism. **(A,B)** Typical example of the 3D reconstruction of serial SEM images of dendritic spines in the middle molecular layer of the hippocampal dentate gyrus from control and T4-treated male **(A)** and female **(B)** mice. Each spine is colored differently. Scale bar: 1 μm. **(C,E)** The mean volume of spines did not show any significant difference between the control and T4-treated groups in male mice (**C**; control, *n* = 167 spines from nine dendrites, three dendrites for each of the three mice; T4, *n* = 247 spines from nine dendrites, three dendrites for each of the three mice) but was decreased in the T4-treated group in female mice (**E**; spine number/9 dendrites; control, *n* = 143 spines from nine dendrites, three dendrites for each of the three mice; T4, *n* = 258 spines from nine dendrites, three dendrites for each of the three mice). Data are presented as mean ± SEM. *P < 0.05 compared to control, Welch’s *t*-test. The same statistical results were obtained with Bootstrap *t*-test. **(D,F)** The spine density (per 1-μm dendrite length) was significantly increased in the T4-treated group in both male **(D)** and female **(F)** mice (*n* = 9 dendrites, three dendrites for each of the three mice). Data are presented as mean ± SEM. *P < 0.05, ***P < 0.001 compared to control, Welch’s *t*-test. N.S., Not significant.

The 3D structures of dendritic spines of granule cells in the middle molecular layer of the hippocampal dentate gyrus were obtained in male and female mice with hyperthyroidism. The 3D reconstruction of serial SEM images allows visualization of the ultrastructure of the dendritic spines in male and female mice (Figures 1A,B). While the mean volume of spines did not show any significant difference between the control (0.064 ± 0.007 μm^3^) and T4-treated groups in male mice (0.061 ± 0.005 μm^3^; Welch’s *t*-test, *t*_(304)_ = 0.2851, *P* = 0.776; Bootstrap *t*-test, ASLboot = 0.763; Figure 1C), a slight decrease in the spine volume was observed in the T4-treated group in female mice from 0.088 ± 0.012 μm^3^ to 0.060 ± 0.005 μm^3^ (Welch’s *t*-test, *t*_(190)_ = 2.206, *P* = 0.029; Bootstrap *t*-test, ASLboot = 0.043; Figure 1E). The spine density in the T4-treated group was significantly increased compared with that in the control group in both male and female mice (Figures 1D,F). In male mice, the spine density increased from 1.969 ± 0.227/μm^3^ (*n* = 9) to 2.929 ± 0.247/μm^3^ (Welch’s *t*-test, *t*_(15)_ = 2.865, *P* = 0.012; Figure 1D). In female mice, the spine density increased from 1.496 ± 0.089/μm^3^ to 2.701 ± 0.196/μm^3^ (Welch’s *t*-test, *t*_(11)_ = 5.597, *P* = 0.0002; Figure 1F). Under control conditions, there were no sex differences in the spine volume (Welch’s *t*-test, *t*_(241)_ = 1.733, *P* = 0.0844; Figures 1C,E) or the spine density (Welch’s *t*-test, *t*_(10)_ = 1.944, *P* = 0.0794; Figures 1D,F). Using the 3D reconstructed images, we also analyzed the spine length and diameter in the control and T4-treated groups in male and female mice. Again, in female mice only, the total length of spine (the head length, but not the neck length) and the head diameter, but not the neck diameter, was significantly decreased in the T4-treated group (Table 1).

**Table 1:** The spine length and diameter in hippocampal granule cells in male and female mice with hyperthyroidism.

	Male			
	Control (*n* = 167)	T4 (*n* = 247)	Welch’s *t*-test	Bootstrap *t*-test
Neck length	0.535 ± 0.026	0.534 ± 0.021	*t*_(346)_ = 0.0075, *p* = 0.9940	ASLboot = 0.997
Head length	0.524 ± 0.017	0.520 ± 0.012	*t*_(320)_ = 0.1923, *p* = 0.8476	ASLboot = 0.854
Total length	1.059 ± 0.034	1.054 ± 0.025	*t*_(331)_ = 0.1039, *p* = 0.9173	ASLboot = 0.927
Neck diameter	0.154 ± 0.009	0.153 ± 0.005	*t*_(286)_ = 0.1471, *p* = 0.8832	ASLboot = 0.881
Head diameter	0.471 ± 0.015	0.473 ± 0.011	*t*_(327)_ = 0.1342, *p* = 0.8933	ASLboot = 0.894
	**Female**			
	**Control (*n* = 143)**	**T4 (*n* = 258)**	**Welch’s *t*-test**	**Bootstrap *t*-test**
Neck length	0.535 ± 0.029	0.475 ± 0.018	*t*_(252)_ = 1.750, *p* = 0.0814	ASLboot = 0.085
Head length	0.588 ± 0.024	0.528 ± 0.012	*t*_(210)_ = 2.310, *p* = 0.0218	ASLboot = 0.022
Total length	1.124 ± 0.036	1.002 ± 0.023	*t*_(256)_ = 2.830, *p* = 0.0050	ASLboot = 0.006
Neck diameter	0.143 ± 0.006	0.132 ± 0.004	*t*_(236)_ = 1.406, *p* = 0.1609	ASLboot = 0.189
Head diameter	0.523 ± 0.022	0.465 ± 0.011	*t*_(214)_ = 2.384, *p* = 0.0180	ASLboot = 0.017

*The spine length and diameter were analyzed in the 3D reconstructed images of dendritic spines presented in Figure 1*.

### Behavioral Tests Show Sex-Dependent Changes in Hyperthyroidism

**Figure 2:**
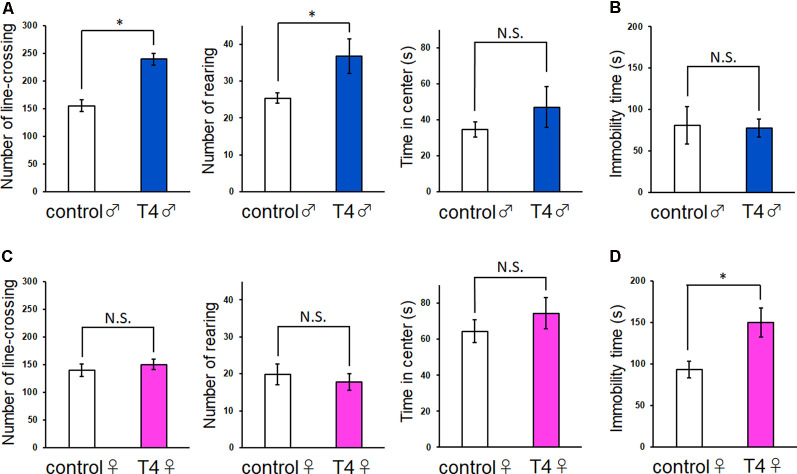
Behavioral tests in male and female mice with hyperthyroidism. **(A,C)** In the open field test, locomotor activity (the number of line-crossing) and the number of rearing were increased in T4-treated male mice (**A**; control, *n* = 5; T4, *n* = 4) but not in female mice (**C**; control, *n* = 5; T4, *n* = 5). The time in the center showed tendency to increase with T4, but was not significantly different in either male or female mice. **(B,D)** In the tail suspension test, the immobility time was not affected in T4-treated male mice (**B**; control, *n* = 5; T4, *n* = 4), but was significantly increased in T4-treated female mice (**D**; control, *n* = 5; T4, *n* = 5). Data are presented as mean ± SEM. *P < 0.05 compared to control, Student’s *t*-test. N.S., Not significant.

In the open field test, the increases in the locomotor activity and rearing behavior were observed in T4-treated male mice (Figure 2A). The number of line crossing of 155.2 ± 11.9 times in control male mice increased significantly to 240.0 ± 13.8 times in T4-treated male mice (Student’s *t*-test, *t*_(7)_ = 4.676, *P* = 0.002). The number of rearing also significantly increased from 25.4 ± 1.5 times in control to 36.8 ± 6.0 times in T4-treated male mice (Student’s *t*-test, *t*_(7)_ = 2.048, *P* = 0.035). The number of grooming was not changed (data not shown). On the other hand, no significant changes in locomotor activity were observed in T4-treated female mice (Figure 2C). The number of line crossings was 140.2 ± 12.7 times in control and 150.2 ± 10.6 times in T4-treated female mice (Student’s *t*-test, *t*_(8)_ = 0.605, *P* = 0.562). The number of rearing was 19.8 ± 3.1 in control and 17.8 ± 2.5 in T4-treated female mice (Student’s *t*-test, *t*_(8)_ = 0.502, *P* = 0.629). The time in the center was not affected by T4 treatment in either male or female mice (Figures 2A,C, right panels), reflecting absence of anxiety-like behaviors. In the tail suspension test, the immobility time in control (80.8 ± 25.1 s) was not affected by T4 treatment (77.3 ± 13.8 s) in male mice (Student’s *t*-test, *t*_(7)_ = 0.115, *P* = 0.912; Figure 2B), while the immobility time significantly increased from 93.2 ± 11.4 s to 150.0 ± 19.8 s in T4-treated female mice (Student’s *t*-test, *t*_(8)_ = 2.491, *P* = 0.038; Figure 2D), suggesting that female mice with hyperthyroidism show depression-like behavior.

## Discussion

### Changes in Dendritic Spine Density and Size in Hyperthyroidism

In this study, the 3D reconstruction of spine structures revealed the increase in dendritic spine density in dentate granule cells in male and female mice with hyperthyroidism. Thyroid hormone likely induces the formation of dendritic spine with normal size in male but of smaller size in female mice, although the possibility that thyroid hormone reduces the size of preexisting spines in female mice cannot be ruled out. In agreement with our results, increased spine density with reduced dendritic branching by T4 administration was reported in CA3 pyramidal neurons of the rat hippocampus (Sala-Roca et al., 2008). Effects of thyroid hormones on dendritic spine density are controversial, and opposing results were reported, for example, the decrease in dendritic spines in CA1 pyramidal cells or dentate granule cells in rat models of hyperthyroidism (Gould et al., 1990; Martí-Carbonell et al., 2012) and in a mouse hypothyroid model with T3 supplementation (Buras et al., 2014). As acute T3 administration is shown to activate microglial phagocytic activity (Mori et al., 2015; Noda et al., 2016), it is possible that activated microglia induce stripping of synapses (Kettenmann et al., 2013), resulting in the reduction of synapse density. Contradicting results may reflect different ways of making animal models. In previous studies, spine morphology was analyzed using Golgi impregnation, and spine volume was not analyzed. To the best of our knowledge, we performed the first study on spine morphology under the state of hyperthyroidism.

The major input to the dentate gyrus is the perforant path axons from the entorhinal cortex; the perforant path makes excitatory synapses onto spines of granule cell dendrites (Kitahara et al., 2016). As the dentate gyrus is known to function as the main gateway to the hippocampus, thyroid hormone-induced increase in dendritic spines density in granule cells may result in the increase in activity of hippocampal neural circuit. Since thyroid hormone accelerates the differentiation of adult hippocampal progenitors (Kapoor et al., 2012), adult born neurons may affect the formation of dendritic spines of mature granule cells in the hippocampal dentate gyrus in hyperthyroidism.

To clarify mechanisms of altered spine structure and the related controversies, more studies need to be done. In particular, transcriptomic analysis in specific cell types as well as proteomic analysis with a different time span of T4 administration is valuable to identify key molecules involved in spine remodeling by thyroid hormone. If cell-type-specific effects of thyroid hormone on gene expression are profiled in the hippocampus and/or prefrontal cortex, the reason for controversial results may be resolved. As a consequence, we may be able to understand what is going on in the brain under hyperthyroidism and design new preventive or therapeutic strategies for depression and anxiety disorder.

### Sex-Dependent Changes in Synaptic Morphology and Behaviors in Hyperthyroidism

As for the sex differences of thyroid function, it seems to exist since early development. The sex difference in the brain thyroid hormone levels during early post-hatching development in zebrafish was reported (Yamaguchi et al., 2017). The onset of the surge of T4 in males preceded that in females, whereas the onset of the surge of T3 in males succeeded that in females. It was also reported that neonatal thyroid hormone administration results in dramatic morphological changes except spine density in adult hippocampal CA3 pyramidal neurons, although no sex differences in magnitude of thyroid hormone-induced changes were detected (Gould et al., 1990). In our study, thyroid hormone induced the formation of spines with normal size in male mice but the formation of smaller spines in female mice, suggesting that thyroid hormone regulates the formation of spine and performant path-granule cell synapse differentially between male and female mice.

Animal behaviors also showed sex differences in the mouse model of hyperthyroidism (Figure 2). Hyperactivity observed in male mice may be related to an increase in the activity of hippocampal neural circuit when exposed to a novel environment under the state of hyperthyroidism. On the other hand, female mice with hyperthyroidism showed depression-like behavior. It is currently unknown whether depression-like behavior in female mice with hyperthyroidism can be attributed to the formation of performant path-granule cell synapse with small spine. We previously reported that antidepressant treatment induces enlargement of dendritic spine of hippocampal granule cells, suggesting that the decrease in spine size of performant path-granule cell synapse may be associated with depression-like behavior.

Depression is thought to be associated with neuronal atrophy including loss of synapses in the prefrontal cortex and hippocampus in humans and rodent chronic stress models, and alterations of spine synapse connectivity likely contribute to behavioral symptoms of depression (Duman and Aghajanian, 2012; Duman and Duman, 2015). Chronic stress is shown to decrease spine density in the hippocampal CA1 and CA3, but an increase or no change in spine density has also been reported (Duman and Duman, 2015; Qiao et al., 2016). Opposite effects of chronic stress on spine density were reported in male and female rodents: a decrease in male but an increase in female (Qiao et al., 2016). This may explain why the increase in spine density in female, but not male, which we observed with hyperthyroidism, displayed depression-like behaviors. Further investigations to directly link thyroid hormone-induced changes of spine density and morphology in neurons in the hippocampus as well as in other brain regions to behavioral changes are therefore needed.

Behaviors of mice with hyperthyroidism were evaluated with the open field and tail suspension tests. However, there is a limitation of two behavioral tests to elucidate differential behavioral effects of hyperthyroidism in male and female mice. It would be ideal to apply a battery of behavioral tests to assess depression-like behavior (e.g., the forced swim test), anxiety-like behavior (e.g., the elevated plus-maze test), and anhedonia-like behavior (e.g., the sucrose preference test).

It is likely that sex hormones such as estrogen and testosterone may contribute to sex differences in neurotransmission, in functional connectivity, and in brain structure (Barth et al., 2015). Transcriptional and translational differences in microglia from male and female brain were reported (Guneykaya et al., 2018), which may or may not be a steady-state influence of sex hormone. We have observed sex difference in glial morphology in adult mice with hyperthyroidism (Noda et al., 2016) as well, which may also contribute to the behavioral changes. It is necessary to elucidate molecular mechanism of the sex differences of neurons and glia under conditions of thyroid dysfunction. From these results, it will become clearer how important it is to control thyroid hormone for brain function and to prevent or treat neurological symptoms related to thyroid dysfunction.

## Data Availability Statement

All datasets presented in this study are included in the article.

## Ethics Statement

The animal study was reviewed and approved by Committee for Animal Care and Use of Kyushu University and Kurume University School of Medicine.

## Author Contributions

TN, MK, and YY designed and conducted experiments, analyzed data, and helped to write the manuscript. YK, MK, KO, TS, and K-iN conducted experiments and analyzed data. TK conducted statistical analysis. AN analyzed data and helped to write the manuscript. MN designed experiments and wrote the manuscript.

## Conflict of Interest

The authors declare that the research was conducted in the absence of any commercial or financial relationships that could be construed as a potential conflict of interest.
